# Subretinal adipose tissue-derived mesenchymal stem cell implantation in advanced stage retinitis pigmentosa: a phase I clinical safety study

**DOI:** 10.1186/s13287-016-0432-y

**Published:** 2016-12-01

**Authors:** Ayse Oner, Z. Burcin Gonen, Neslihan Sinim, Mustafa Cetin, Yusuf Ozkul

**Affiliations:** 1 Department of Ophthalmology, Erciyes University, Kayseri, Turkey; 2 Genome and Stem Cell Center (GENKOK), Erciyes University, Kayseri, Turkey; 3 Department of Oral and Maxillofacial Surgery, Faculty of Dentistry, Erciyes University, Kayseri, Turkey; 4 Department of Internal Medicine, Division of Hematology, Faculty of Medicine, Erciyes University, Kayseri, Turkey; 5 Department of Medical Genetics, Faculty of Medicine, Erciyes University, Kayseri, Turkey

**Keywords:** Adipose tissue-derived mesenchymal stem cell, Retinitis pigmentosa, Subretinal implantation

## Abstract

**Background:**

This prospective clinical case series aimed to investigate the safety of subretinal adipose tissue-derived mesenchymal stem cell (ADMSC) implantation in advanced stage retinitis pigmentosa (RP).

**Methods:**

This study included 11 patients with end-stage RP who received subretinal implantation of ADMSCs. All patients had a total visual field defect and five of them only had light perception. The best corrected visual acuity (BCVA) in the study was 20/2000. All patients had undetectable electroretinography (ERG). The worst eye of the patient was operated on and, after total vitrectomy with a 23 gauge, ADMSCs were injected subretinally. Patients were evaluated at day 1, at weeks 1–4, and then once a month for 6 months, postoperatively. BCVA, anterior segment and fundus examination, color photography, and optical coherence tomography (OCT) were carried out at each visit. Fundus fluorescein angiography (FFA), perimetry, and ERG recordings were performed before treatment and at the end of month 6, and anytime if necessary during the follow-up.

**Results:**

All 11 patients completed the 6-month follow-up. None of them had systemic complications. Five patients had no ocular complications. One of the patients experienced choroidal neovascular membrane (CNM) at the implantation site and received an intravitreal anti-vascular endothelial growth factor drug once. Five patients had epiretinal membrane around the transplantation area and at the periphery, and received a second vitrectomy and silicon oil injection. There was no statistically significant difference in BCVA and ERG recordings from baseline. Only one patient experienced an improvement in visual acuity (from 20/2000 to 20/200), visual field, and ERG. Three patients mentioned that the light and some colors were brighter than before and there was a slight improvement in BCVA. The remaining seven patients had no BCVA improvement (five of them only had light perception before surgery).

**Conclusions:**

Stem cell treatment with subretinal implantation of ADMSCs seems to have some ocular complications and should be applied with caution. The results of this study provide the first evidence of the short-term safety of ADMSCs in humans, and clarifies the complications of the therapy which would be beneficial for future studies. To optimize the cell delivery technique and to evaluate the effects of this therapy on visual acuity and the quality of life of these patients, future studies with a larger number of cases will be necessary.

## Background

Retinitis pigmentosa (RP) is a group of inherited retinal disorders characterized by progressive loss of photoreceptors. The disease initially begins by night blindness, and progresses to tunnel vision and eventual loss of central vision, ending with total blindness. Up to now, there is no curative treatment for this retinal disease. New approaches for RP therapy include restoring defective genes and stem cell transplantation to replace defective or dead cells [1–5].

Stem cells are undifferentiated cells which have the ability to self-renew and differentiate into mature cells. They are highly proliferative, implying that an unlimited number of mature cells can be generated from a given stem cell source. On this basis, cell replacement therapy has been evaluated in recent years as a viable alternative for various pathologies. This therapy hypothesizes that new retinal cells could be generated from stem cells to replace the damaged cells in the diseased retina. This theory is mainly established from embryonic stem cells (ESCs) and induced pluripotent stem cells (IPSCs). In addition, stem cells (particularly mesenchymal stem cells (MSCs)) are able to perform multiple functions, such as immunoregulation, anti-apoptosis of neurons, and neurotrophin secreting. MSCs are an adult stem cell population of stromal progenitor cells of mesodermal origin which were originally identified in the bone marrow. They can also be found in other body systems such as adipose tissue, liver, umblical cord, central nervous system, and dental tissues [2–5]. Many studies suggested that MSCs are able to maintain and regulate the microenvironment in different models of retinal degeneration [6–10]. Experimental studies also reported that MSCs have the potential to differentiate into retinal progenitor cells, photoreceptors, and retinal neural-like cells [6, 7].

With the progress in basic medical sciences, several phase I/II clinical trials were approved by the Food and Drug Administration (FDA), and recently we have seen the promising and encouraging results of some of these stem cell clinical trials [8–13].

In this study, we aimed to investigate the safety of subretinal adipose tissue-derived mesenchymal stem cell (ADMSC) implantation in advanced-stage RP.

## Methods

This single-center, prospective, phase I clinical safety study enrolled 11 patients with irreversible vision loss from RP who were seen in the Retina and Vitreous Section of the Department of Ophthalmology in our University. The study was performed in accordance with the Declaration of Helsinki and was approved by the Institutional Review Board (Review number for approval: 2014/390). It was also approved by the Review Board of Stem Cell Applications within the Ministry of Health in accordance with the regulations in our country (Review number for approval: 56733164/203). All patients were instructed about the objectives and methodology of the study and gave written informed consent to participate.

### Patient eligibility

There are limited data about clinical applications of MSCs and the safety of this therapy still needs to be clarified. Therefore, the study included patients with end-stage RP who are considered as legally blind.

Patients were included if they had: 1) a diagnosis of hereditary retinal dystrophy classified clinically as RP; 2) an Early Treatment Diabetic Retinopathy Study best corrected visual acuity (BCVA) worse than 20/200; 3) a visual field less than 20 degrees, considered as legally blind; 4) decreased electroretinography (ERG) recordings; and 5) were aged older than 18 years.

Exclusion criteria were: 1) previous ocular surgery other than cataract extraction; 2) presence of a cataract or other media opacity that would prohibit high-quality ocular imaging or that would affect ERG or visual field evaluation; 3) presence of other ophthalmic disease, such as glaucoma, uveitis, strabismus, or nystagmus; 4) any other systemic disease such as neurological disease that would affect the results. If both eyes were eligible for treatment, the eye with the worst visual acuity was used in the study.

The demographic data of the patients were collected and ophthalmic evaluations and surgical procedures were performed by a single retina specialist (AO). Fundus photography, fundus fluorescein angiography (FFA), optical coherence tomography (OCT), perimetry, and ERG recordings were performed by the same experienced ophthalmic technician.

All patients had a complete ophthalmic examination before surgery. BCVA was measured with the Snellen chart. All patients underwent fundus photography and FFA using the Zeiss FF 450 (Carl Zeiss Meditec AG, Germany). OCT was performed using the Heidelberg OCT Device (Heidelberg Engineering, Heidelberg, Germany) with a standardized scanning protocol. Visual field examination was performed by Octopus Goldmann Perimetry (Octopus 900, Haag Streit İnternational, Switzerland). ERG tests were recorded with Tomey Primus 2.5 (Tomey GmbH, Erlangen, Germany) in accordance with the guidelines of the International Society for Clinical Electrophysiology of Vision (ISCEV) [14].

### Isolation and culture of ADMSCs

To eliminate donor-based differences, ADMSCs obtained from the adipose tissue of a single donor were used for all patients in this study. Subcutaneous adipose tissue was carried to the laboratory in a transfer solution of Dulbecco’s modified Eagle medium (DMEM) with 1 g/l d-glucose (low glucose; Biological Industries, Kibbutz Beit Haemek, Israel) containing 2% penicillin-streptomycin solution (10,000 units/ml penicillin G sodium salt, 10 mg/ml streptomycin sulfate; Biological Industries). Adipose tissue was washed in phosphate-buffered saline (PBS; Biological Industries) three times and cut into small pieces and then digested with 2.5 mg/ml collagenase type II (Sigma-Aldrich, Taufkirchen, Germany) for 30 min at 37 °C to generate single-cell suspension. The digested tissues were filtered through a 70-μm cell strainer and centrifuged at 350 *g* for 5 min to obtain a pellet. The pellet was resuspended in DMEM-based media containing 10% human serum, 1% penicillin-streptomycin solution, and 1% stable glutamine (Biological Industries) and cultured at 37 °C under 5% CO_2_. After 3–4 days of maintenance, the culture medium was removed to eliminate the non-attached cell fraction. The medium was replaced twice a week. The culture medium was changed after reaching 80–85% confluence, and the cells were detached with 0.25% trypsin EDTA solution C (0.05%) and EDTA (0.02%) (Biological Industries). The cells were collected, centrifuged at 350 *g* for 5 min, and expanded to the required duplication. ADMSCs were then harvested and cryopreserved until use. Before the appointed surgery date, sufficient cryopreserved vials were thawed to provide the required dose for administration. The frozen ADMSCs were thawed and cultured under the same conditions. ADMSCs were recovered, washed with PBS and trypsin/EDTA, and then resuspended in saline solution and transferred to the surgery room in a temperature-controlled bag within 1 h. The total injection volume was 2.47 × 10^6^ ± 0.11/150 μl per patient for this study. The procedure for ADMSC preparation was performed under good manufacturing practice (GMP) conditions in the Genome and Stem Cell Center of our University. All of the donation, manufacturing, and testing procedures were carried out according to GMP protocols authorized by the Ministry of Health in our country. For release testing, ADMSCs were assessed for cell appearance, viability, identification, purity, content, and potency. In addition, ADMSCs were screened for contamination.

For determining the potency, the suppression effect of MSCs on lymphocytes was studied. A peripheral blood sample was taken from the healthy donor and peripheral blood mononuclear cells (PBMCs) were collected by density gradient centrifugation using lymphocyte separation medium (LSM; Biological Industries, BI #01-899-U04). Then PBMCs were incubated at 37 °C in DMEM culture medium containing 10% human serum, 1% l-glutamine and 1% penicillin-streptomycin. PBMCs were stimulated with 1% phytohemagglutinin (PHA-P; Sigma, #L1668) and the effect of MSCs on lymphocyte proliferation was studied. MSCs (5 × 10^4^) were cultured with PBMCs (5 × 10^5^) for 48 h, and 0.5 mg/ml MTT was added. The entire medium was aspirated, and 100 μl DMSO was added to dissolve formazan crystals after 3 h. Dissolved formazan crystals were read spectrophotometrically at 570 nm. The percentage inhibition of lymphocyte proliferation was determined.

### Flow cytometry analyses

ADMSCs were subjected to flow cytometry analyses for confirmation that ADMSCs maintain their phenotypic characteristics in vitro. After the third passage, cells were harvested, centrifuged, and resuspended in PBS at a minimum concentration of 1 × 10^6^ cells/ml. Immunophenotyping characterization of ADMSCs was performed with antibodies against the following combination of human antigens: CD11b, CD19, CD34, CD44, CD45, CD73, CD90, and CD105 (BD Stem Flow hMSC kit, BD Cat. No: 562245). Flow cytometry analyses were performed using Navios (Beckman Coulter, USA) before application to all patients in this study. The data were analyzed with KALUZA software (Beckman Coulter).

### Subretinal implantation of MSCs

All patients received a routine 23 gauge pars plana vitrectomy operation with retrobulber anesthesia under sterile conditions. Vitrectomy was carried out using the Dutch Ophthalmic Research Center EVA (DORC, Zuidland, Netherlands) system. After core vitrectomy, posterior vitreous detachment was induced with the assistance of triamcinolone acetonide and total vitrectomy was completed. An area called a transition zone (the zone between the affected and relatively unaffected retinal area) far from the fovea was selected for the subretinal implantation of ADMSCs. Subretinal ADMSCs were injected at a concentration of 2.47 × 10^6^ ± 0.11/150 μl with a 41-gauge needle. Fluid-air exchange was performed at the end of surgery. No laser treatment was performed. All surgery was carried out under local anesthesia by a single surgeon (AO).

#### Modified technique

The modified technique focused on the removal of the whole vitreous gel and the clearance of the scattered epiretinal stem cells. To shave the peripheral vitreous, 360° scleral indentation was performed cautiously. The triamcinolone acetonide assistance was repeated to visualize the residual vitreous fibers, posterior hyaloid remnants, or preretinal membranes. To decrease the number of scattered epiretinal stem cells, fluid-air exchange was applied at least three times after the subretinal implantation procedure.

### Postoperative follow-up

Patients were hospitalized at least 1 day after surgery and they are instructed to maintain a face-down position to induce the subretinal spread of MSCs during the first postoperative day. Ophthalmological evaluations were performed at day 1, at weeks 1–4, and then once a month for 6 months after the surgery. BCVA, anterior segment and fundus examination, color photography, and OCT were carried out at each visit. FFA, perimetry, and ERG recordings were performed before surgery and at the end of month 6, and anytime if necessary during the follow-up. The patients received topical steroid and antibiotic eye drops four times a day for 2 months. Low-dose systemic cyclosporin A (2.5–3 mg/kg/day, twice daily) was used as an immunosuppressive agent for 2 months starting from 1 week before surgery.

The primary outcome measures were the incidence and severity of ocular and systemic adverse events associated with ADMSC treatment.

## Results

### Morphology and phenotype of culture-expanded ADMSCs

**Fig. 1:**
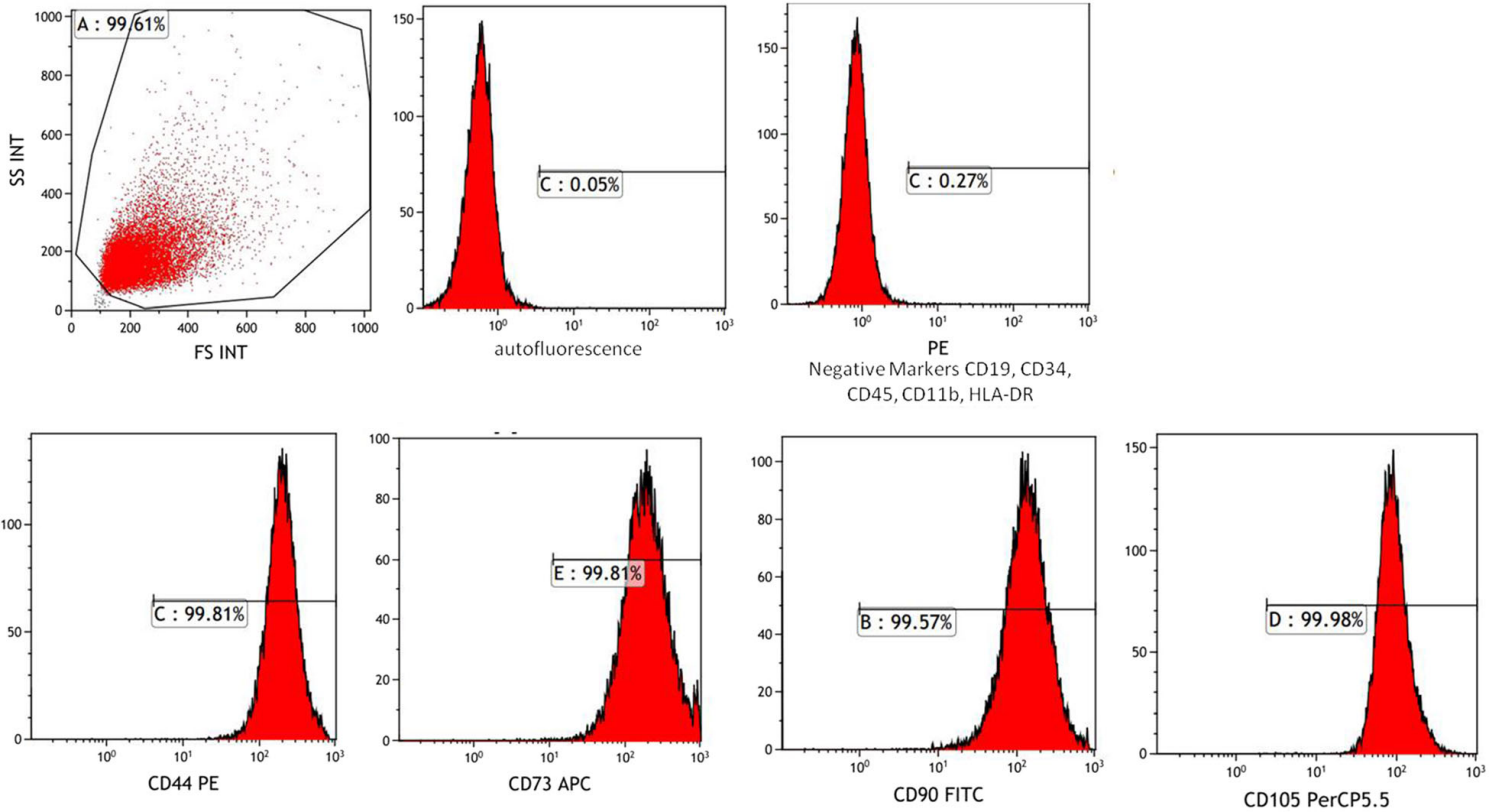
Immunophenotypic characteristics of ADMSCs. Surface marker expressions are represented as percentage expression levels

ADMSCs were spindle-shaped cells with a fibroblast-like morphology, and they attached to the plate during cell culture. These cells were found to be efficient according to the inhibition of lymphocyte proliferation before release. For immunophenotypic characterization of ADMSCs, culture-expanded cells at the third passage were examined for surface protein expression using flow cytometry. The ADMSCs were positive for CD44, CD73, CD90, and CD105, and negative for CD11b, CD34, CD45, and HLA-DR; the results of the analysis are listed in Table 1 and Fig. 1. No evidence of bacterial or fungal contamination was observed in the cells which were tested before release. Cell viability evaluated by trypan blue exclusion was >90.3 ± 0.5% before cell transplantation.

**Table 1:** Immunophenotype of culture-expanded human adipose-derived mesenchymal stem cells

Surface markers	Positive percentage (%) (*n* = 11)
CD45	0.5 ± 0.3
CD34	0.5 ± 0.3
CD11b	0.5 ± 0.3
CD19	0.5 ± 0.3
CD105	99.73 ± 0.1
CD73	99.03 ± 0.7
CD90	94.83 ± 0.5
CD44	99.46 ± 0.6
HLA-DR	0.5 ± 0.3

Data are expressed as mean ± standard deviation

Table 2 shows the demographic and clinical features of the 11 study patients who enrolled in this study. All completed the 6-month follow-up period. The mean age of the subjects was 38.2 years (age range 26–57 years). All subjects had advanced central and peripheral vision loss in both eyes from RP, which was diagnosed by the presence of bone spicules on fundus examination and a flat unrecordable ERG in both eyes. Five of the subjects had only light perception in both eyes for 5 years or longer (range 5–10 years).

**Table 2:** Demographic and visual acuity results according to the Snellen equivalent of enrolled subjects

No./age (years)/sex/eye	Baseline BCVA	Best follow-up BCVA	6-month BCVA
1/44/male/OD	LP	LP	LP
2/57/male/OD	LP	LP	LP
3/42/female/OS	LP	LP	LP
4/34/male/OS	20/20000	20/20000	20/20000
5/34/male/OS	20/2000	20/400	20/2000
6/30/female/OD	20/20000	20/2000	20/2000
7/29/male/OS	20/20000	20/2000	20/2000
8/47/male/OS	LP	LP	LP
9/26/male/OD	20/20000	20/400	20/400
10/46/female/OS	LP	LP	LP
11/32/male/OS	20/20000	20/20000	20/20000

*BCVA* best corrected visual acuity, *LP* light perception, *RE* Right eye, *LE* Left eye

### Follow-up BCVA and eye examination

All 11 patients completed the 6-month follow-up and none of them had systemic complications. Five patients had no ocular complications due to the stem cell treatment. One of the patients experienced choroidal neovascular membrane (CNM) at the implant site and received an intravitreal anti-vascular endothelial growth factor (VEGF) drug once. The first five patients to be operated on had epiretinal membrane (ERM) around the implant area and at the periphery, with localized peripheral tractional retinal detachment at the periphery which required a second vitrectomy and silicon oil injection within the second month. This complication is thought to be due to the vitreal reflux or inadvertent preretinal injection and unwanted preretinal proliferation of MSCs. To prevent the occurrence of ERM, the operation technique was modified as described in the methods section. This modification inhibited ERM formation in the remaining six patients.

There was no statistically significant difference in visual acuity (VA) from baseline. Only one patient experienced VA improvement (from 20/2000 to 20/200). Three patients mentioned that the light and some colors were brighter than before and there was a slight improvement in VA (See Table 2). The remaining seven had no VA improvement (five of them only had light perception before treatment).

Two of the patients experienced a mild vitreus hemorrhage after surgery which cleared in the first week. There were no signs of intraocular inflammation in the study eyes on serial eye examinations. We found no evidence of adverse proliferation, rejection, or serious ocular or systemic safety issues related to the implanted stem cells.

### Perimetry

**Fig. 2:**
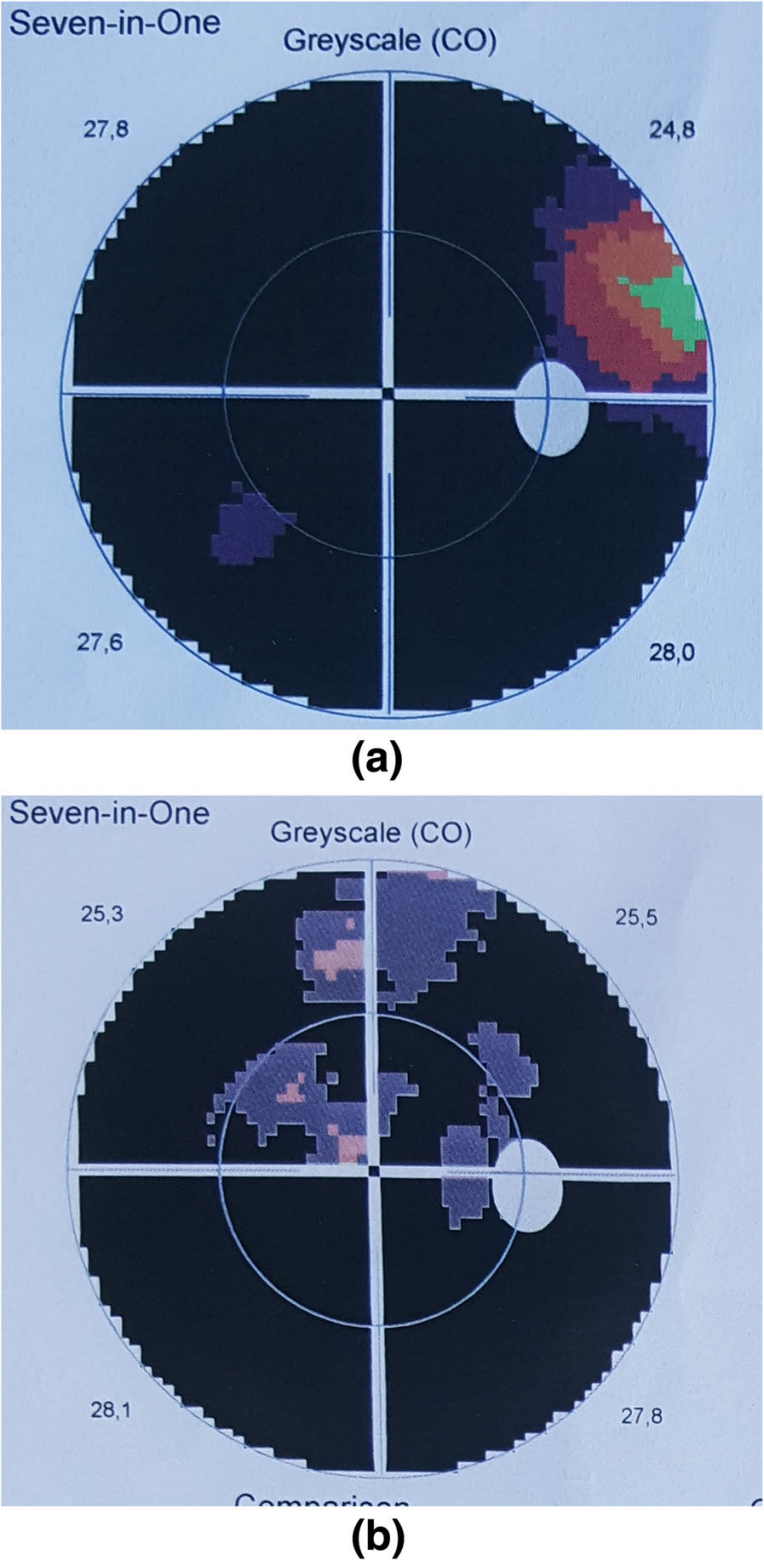
Perimetry results for subject 9 before treatment (**a**) and after treatment (**b**). Note the improvement in visual field

Subject 9 was noted to have improvement in the visual field on Goldmann perimetry at 1-month follow-up examination, which appeared sustained at the final 6-month follow-up visit (Fig. 2). The remaining patients did not show any improvement regarding the perimetry.

### Electroretinography

**Fig. 3:**
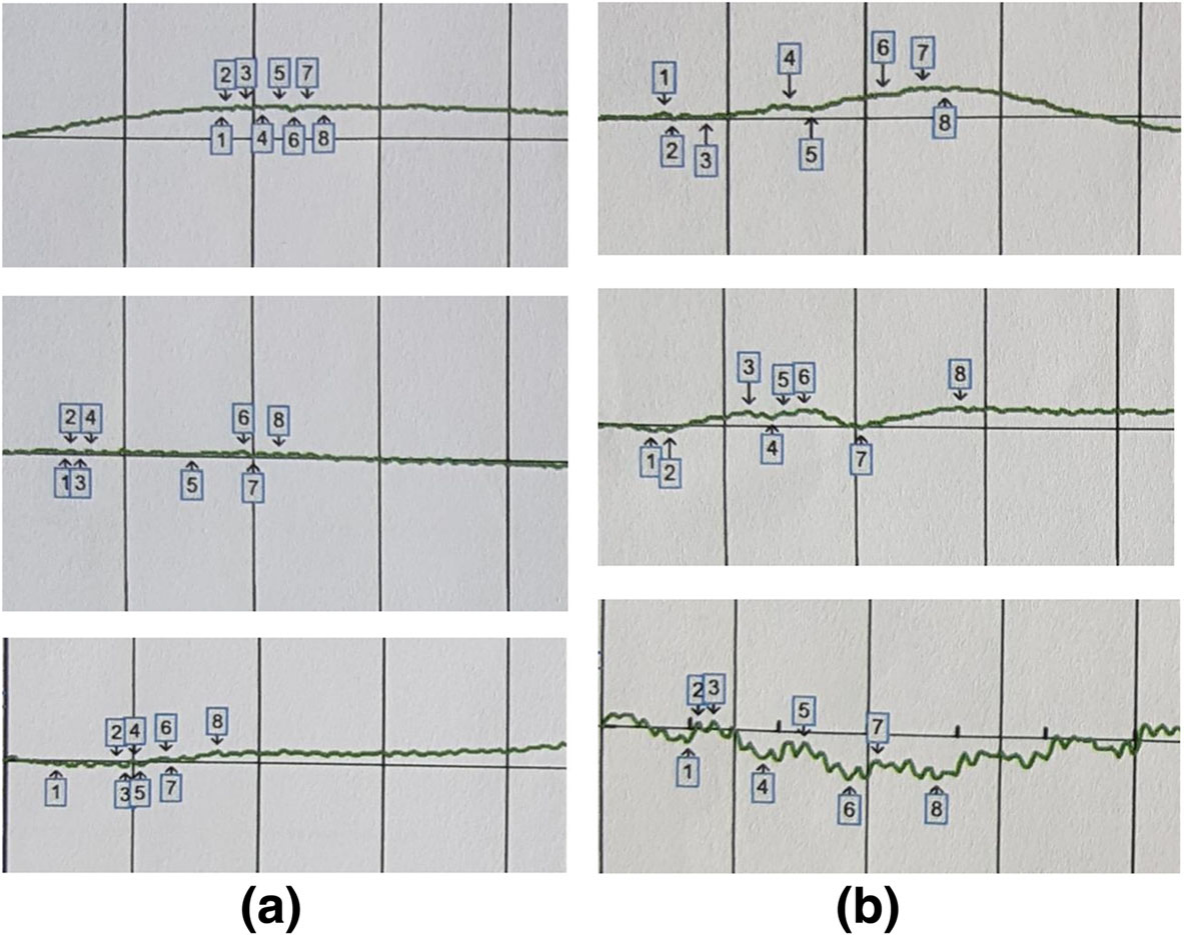
ERG results of subject 9. Rod, maximal combined, and flicker responses before treatment (**a**) and after treatment (**b**). Before treatment, all of the waves were flat; there was a slight improvement in ERG recordings after treatment

Ten subjects had flat unrecordable full-field ERG at baseline and at 6-month follow-up in both eyes. There was a slight improvement in the ERG recordings of subject 9 (Fig. 3).

### Fluorescein angiography

**Fig. 4:**
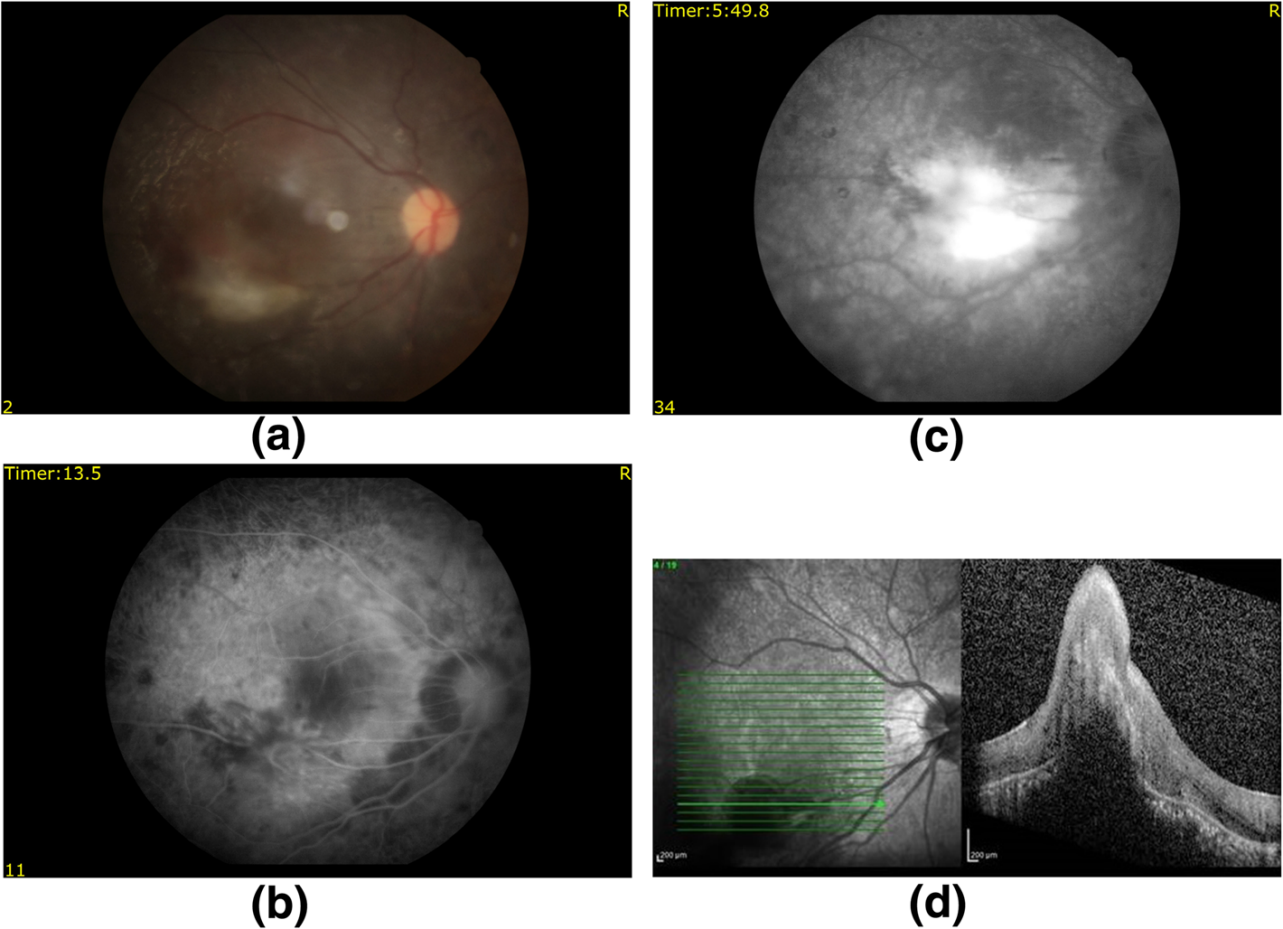
FFA results for patient 6. Color fundus showing hemorrhage and CNM (**a**), FFA images including early (**b**) and late (**c**) phases showing an increase in hyperfluorescence, and OCT image showing elevation and intraretinal fluid (**d**) due to CNM

**Fig. 5:**
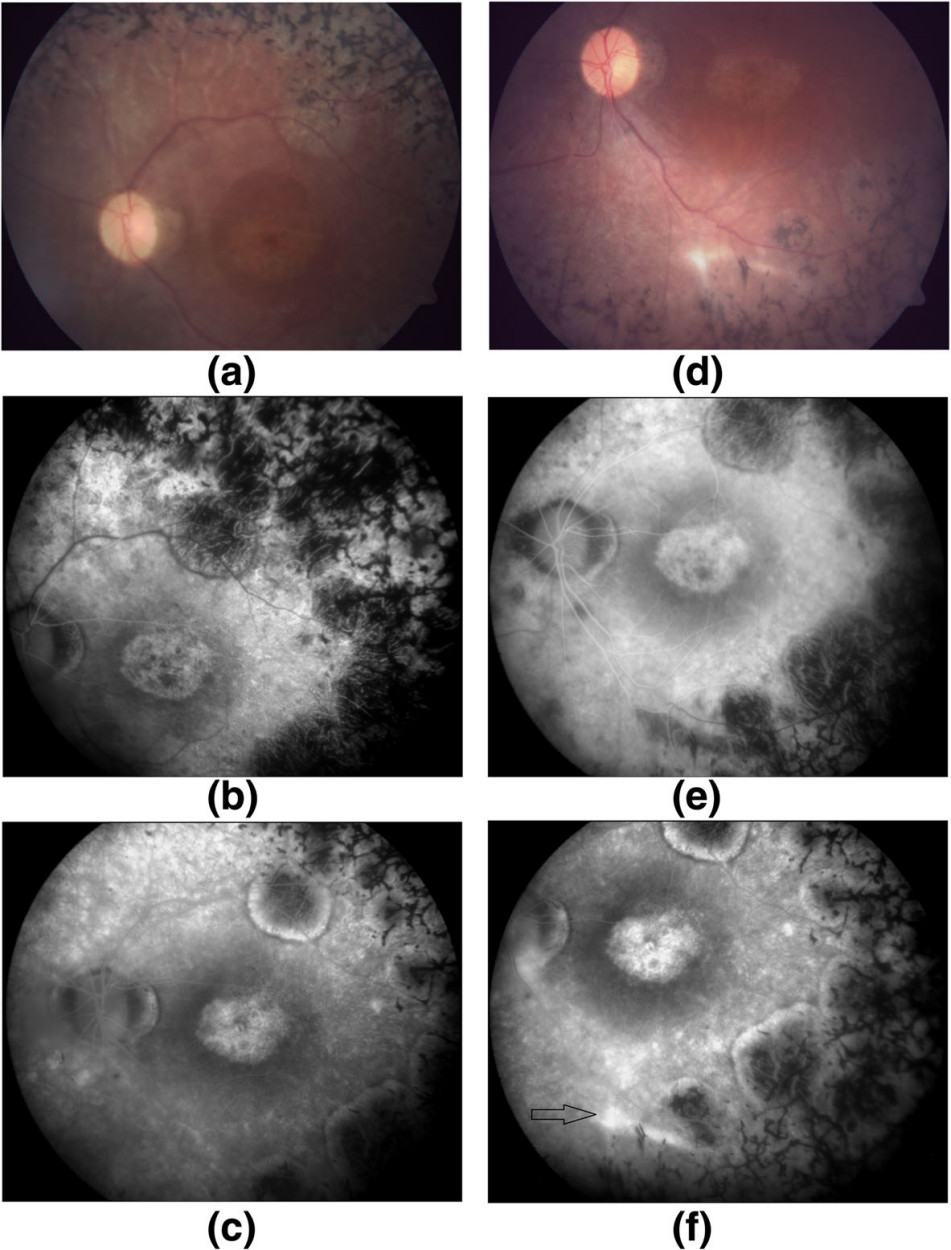
FFA results for patient 7. Baseline color fundus (**a**), early phase (**b**) and late phase (**c**) FFA photos of subject 7, and 6-month color fundus (**d**), early phase (**e**) and late phase (**f**) FFA photos of the same patient. There was staining due to the subretinal scar tissue in the implantation area in the late phase angiogram (*black arrow*)

The FFA of patient 6 showed CNM at month 2 after treatment (Fig. 4). The remaining ten patients who underwent ADMSC implantation showed no signs of fluid collection, edema, or persistent leakage on FFA (Fig. 5).

### OCT Findings

**Fig. 6:**
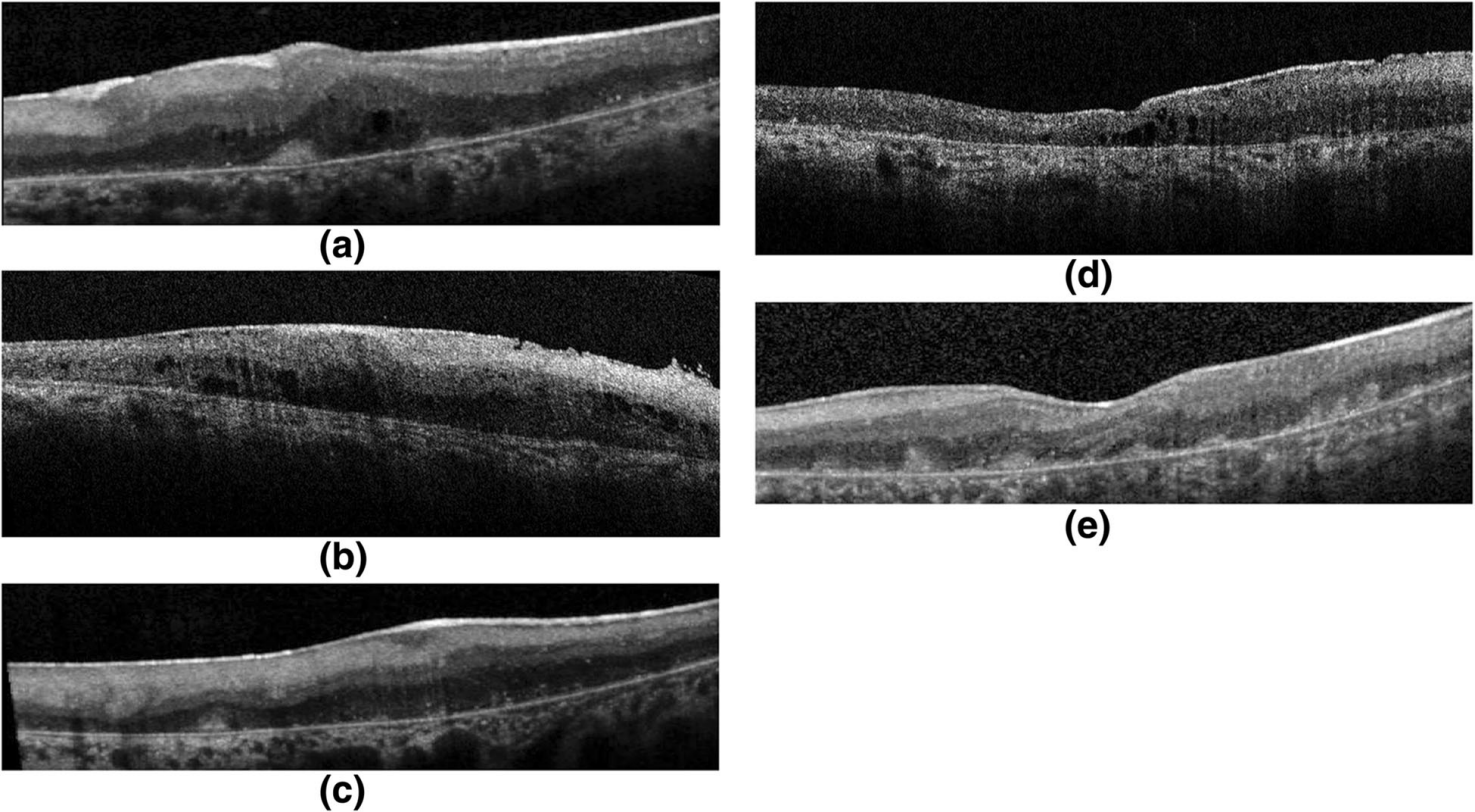
OCT images of five patients (**a**–**e**) demonstrated ERM formation with distortion of the underlying inner retina and varying degrees of retinal edema. There were also varying degrees of cell accumulation on the inner aspects of Bruch’s membrane

**Fig. 7:**
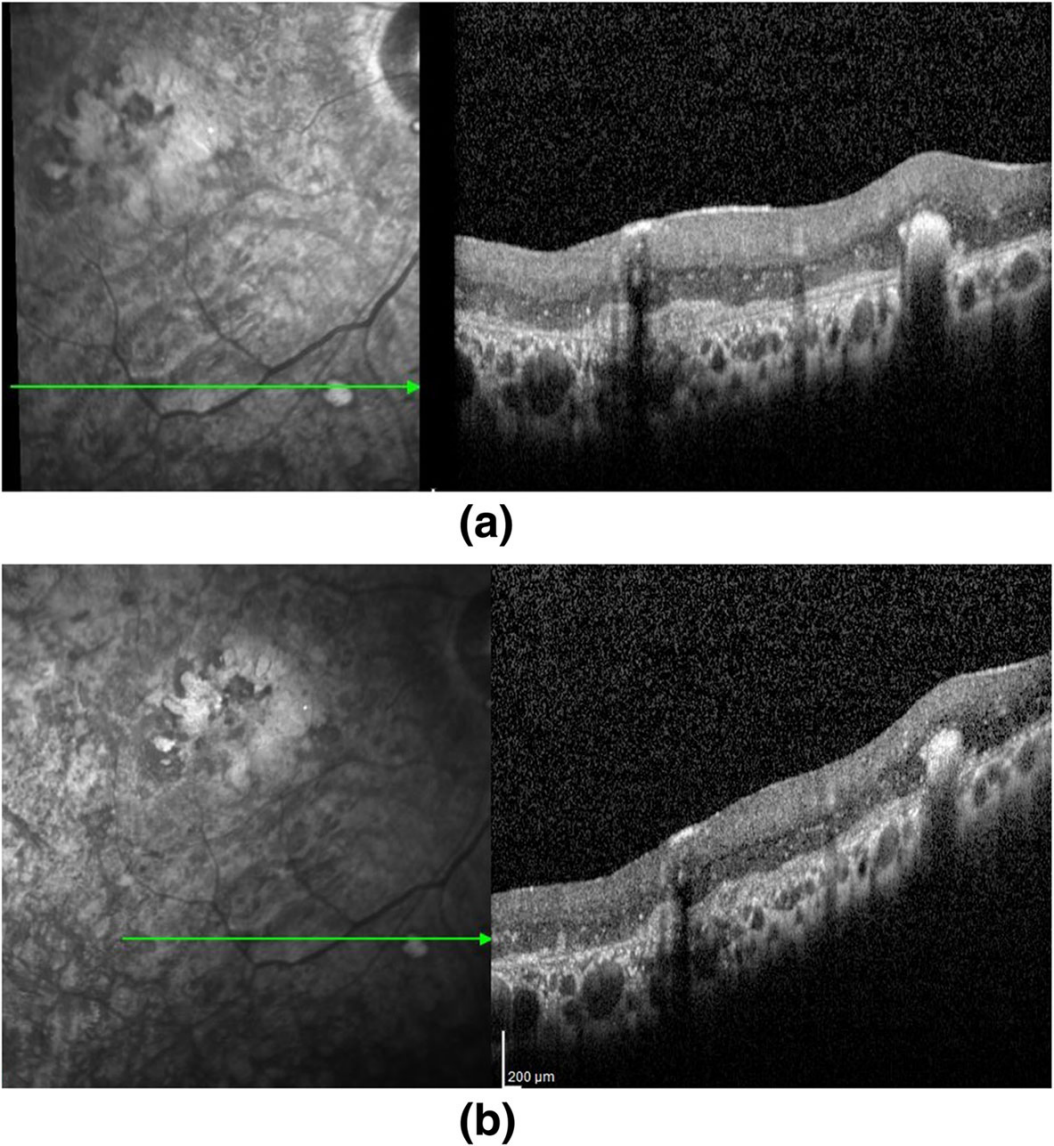
OCT images of subject 9 at month 1 (**a**) and month 6 (**b**). Note the decreasing number of hyper-reflective dots within the retina

OCT images of five patients demonstrated ERM formation with distortion of the underlying inner retina, and varying degrees of retinal edema (Fig. 6). We observed varying degrees of cell accumulation on the inner aspects of Bruch’s membrane after surgery on OCT in all patients, which decreased in size and number during the follow-up period. All patients showed varying number of white dots within the retina during the follow-up period. (Fig. 7)

## Discussion

MSCs are characterized by a high proliferation potential and are able to differentiate into cells of mesodermal, ectodermal, and endodermal origin. Adipose tissue can be obtained using simple procedures such as liposuction performed under local anesthesia, and the number of MSCs in adipose tissue is relatively large. The isolated stem cells are easily grown in culture, and they preserve their properties over many passages. These advantages make adipose tissue an attractive alternative source of stem cells [2, 3, 5, 15, 16].

ADMSCs are multipotent and can differentiate into various cell types, including osteocytes, adipocytes, vascular endothelial cells, cardiomyocytes, pancreatic β-cells, and hepatocytes [17]. An increasing number of studies also report that MSCs are capable of giving rise to neuron-like cells. They are not only able to differentiate into neurons for cell replacement therapy but they also show paracrine effects by modulating the plasticity of damaged host tissues. These cells are able to secrete neurotrophic and survival-promoting growth factors, restore synaptic transmitter release, integrate into existing neural and synaptic networks, and re-establish functional connections [18–21]. In an experimental ocular hypertension model after intravitreal transplantation, MSCs showed a neuroprotective effect in rats [20]. These paracrine activities have not been reported in ESCs or IPSCs. Moreover, MSCs possess strong immunosuppressive properties and inhibit the release of pro-inflammatory cytokines [22]. This allows autologous, as well as allogeneic, transplantation of MSCs. In addition, there is no reported teratoma formation, and there are no moral objections or ethical controversies involved in their application [15, 16]. These advantageous properties lead to the idea of experimental and clinical applications of MSCs to treat different human diseases.

Subretinal injection or transplantation of MSCs has been reported to protect and rescue degenerative retina in retinal degeneration rat models [6, 23]. An experimental study based on paracrine effects reported that the cultured rat MSCs could secrete nerve growth factor (NGF), and that NGF or NGF-neutralizing antibody could activate Müller cell de-differentiation both in vivo and in vitro [24]. In another experimental model, several proteins secreted by human ADMSCs, such as the tissue inhibitor of metalloproteinase-1 and the secreted protein acidic and rich in cysteine, have been shown to protect the retina against light-induced damage in vitro and in vivo [25]. Another study identified a factor called progranulin as a major secreted protein of ADMSCs which showed protective effects against retinal damage both in vitro and in vivo. These findings suggest that ADMSCs have neuroprotective effects in the light-induced retinal damage model [26].

Studies have also confirmed that MSCs are capable of differentiating into different types of retinal cells. A study by Huang et al. [27] showed that MSCs could differentiate into retina pigment epithelium (RPE)-like cells with similar morphological and phagocytic capabilities using the photoreceptor outer segments and RPE conditioned medium. In addition, under certain conditions, MSCs can be further differentiated when transplanted into damaged retina, thereby replacing damaged retinal cells [27, 28]. The early signs of MSC differentiation into retinal progenitor cells were observed at 24 h of co-culture, and the early differentiated retinal lineage cells appeared at 72 h (neurons, rods, Müller cells, retinal ganglion cells, and retinal pigmented epithelial cells). These changes increased during further culture [29]. Another study including in vitro culture and expansion of bone marrow-derived MSCs in rats (rMSCs) showed that rMSCs could differentiate into retinal neural-like cells [30]. In an experimental study by Castanheira et al. [31] MSCs were injected into the vitreous cavity of laser damaged retina. After 8 weeks, they found that the majority of MSCs migrated to the ganglion cell layer, the inner nuclear layer, and the outer nuclear layer, and these migrated cells expressed markers of photoreceptor cells, bipolar cells, amacrine cells, and Müller glial cells [31]. In a recent experimental study by Li et al. [32], subretinal transplantation of human ADMSCs into rats significantly improved the visual function 2 weeks after the transplantation, and this therapeutic effect persisted up to 8 weeks after treatment. They concluded that this therapy effectively delayed the retinal degeneration, enhanced the retinal cell survival, and improved the visual function, and that this was mainly due to ADMSC-dependent anti-apoptotic and neuroprotective effects through its secretion of growth and neurotrophic factors including VEGF [32]. The survival of human ADMSCs for a period of 90 days in the rat vitreous humor and an even longer period of up to 6 months in other eye tissues makes them a promising source to be considered in the regenerative treatment of eye diseases [33].

The eye has numerous advantages for developing stem cell therapies as all tissues of the eye are surgically accessible, and transplanted cells can be monitored. Moreover, the ocular immune privilege might greatly simplify immunosuppressive treatment after transplantation.

After the encouraging results of experimental studies, clinical trials of stem cell transplantation have begun recently in the USA and Europe. In 2012, Schwartz et al. [11] reported preliminary safety data of human ESCs on one dry age-related macular degeneration (AMD) patient and one Stargardt macular dystrophy (SMD) patient. The subretinal transplanted human ESC-derived RPE cells showed no signs of hyperproliferation, tumorigenicity, ectopic tissue formation, or apparent rejection after 4 months. The 22-month follow-up data of this study included nine dry AMD and nine SMD patients. BCVA improved in ten eyes, improved or remained the same in seven eyes, and decreased by more than ten letters in one eye, whereas the untreated fellow eyes of the patients did not show similar improvements in VA. The results of this study provide the first evidence of the medium-term to long-term safety, graft survival, and possible biological activity of pluripotent stem cells in individuals with retinal disease [12].

In another clinical study with human ESC-derived RPE cells [13], the authors reported the safety and tolerability of subretinal transplantation in four Asian patients: two with dry AMD and two with SMD. The patients were followed for 1 year and there was no evidence of adverse proliferation, tumorigenicity, ectopic tissue formation, or other serious safety issues related to the transplanted cells. Visual acuity improved 9–19 letters in three patients and remained stable in one patient. These results confirmed that human ESC-derived cells could serve as a potentially safe new source for regenerative medicine [13].

In a prospective, phase I study, including three patients with RP and two patients with cone-rod dystrophy, Siqueira et al. [8] evaluated the short-term safety of a single intravitreal injection of autologous bone marrow-derived MSCs and they reported no detectable structural or functional toxicity to the retina over a period of 10 months. They measured a one-line improvement in BCVA in four patients 1 week after injection, and this improvement was maintained throughout follow-up [8]. The follow-up data of this study included 20 patients with RP who received intravitreal bone marrow-derived MSCs. They evaluated the vision-related quality of life of patients using the National Eye Institute Visual Function Questionnaire-25 (NEI VFQ-25) before treatment and 3 and 12 months after treatment. There was a statistically significant improvement in the quality of life of patients 3 months after treatment, whereas by month 12 there was no statistically significant difference from baseline [9].

In a recent study by Park et al. [10], six subjects (six eyes) with irreversible vision loss from retinal vascular occlusion, hereditary or non-exudative AMD, or RP received a mean of 3.4 million intravitreal bone marrow-derived MSCs. The therapy was well tolerated with no intraocular inflammation or hyperproliferation; BCVA and full-field ERG showed no worsening after 6 months, and they concluded that intravitreal autologous bone marrow-derived MSC cell therapy appeared feasible.

To the best of our knowledge, ours is the first clinical study including the results of subretinal implantation of ADMSCs in RP patients. The purpose of this study was to determine the safety and tolerability of this therapy. We found no serious systemic or ocular adverse events in these 11 subjects. Ophthalmological examinations, including visual acuity, perimetry, color fundus photography, FFA, OCT, and ERG, did not identify any significant safety concerns after surgery.

The first five patients to be operated on developed ERMs and localized peripheral tractional retinal detachments which required a second surgery. This is relatively high compared with the rate of ERM development after the vitrectomy procedure. We thought that this complication may have arisen from inadvertent preretinal injection of cells or reflux of transplanted cells from the subretinal space. With the idea that rinsing the epiretinal area may help to decrease the number of scattered epiretinal stem cells, the technique was modified and fluid-air exchange was applied at least three times after the subretinal implantation procedure.

Vitreous abnormalities have been well documented in all RP stages. In the advanced stage of the disease, an atypical posterior vitreous detachment, posterior vitreoschisis, and formation of condensed irregular fiber aggregates could be found in RP patients [34–36]. These abnormalities could cause some difficulties in the removal of posterior vitreous which may lead to the ERM formation found in our study. In our modified technique, we paid extreme attention to shave the peripheral vitreous, and to visualize the residual posterior vitreous, posterior hyaloid remnants, or preretinal membranes and remove them. This modification prevented the development of ERM in the remaining six patients.

ERM formation was also reported in the study by Song et al. in two of the four patients who received subretinal human ESC-derived RPE cells [13]. They reported that ERM developed at 2 weeks in both patients and caused minimal distortion of the underlying inner retina which did not require a second surgery [13].

During the follow-up period, CNM developed in one of our study patients in the MSC transplanted eye at 8 weeks which was successfully treated with anti-VEGF drugs. The location of the CNM was primarily in the bleb area. Song et al. [13] also reported a CNM formation in one of four patients who received three monthly Lucentis injections. This complication may have developed due to trauma to the Bruch’s membrane during the surgical procedure. Another possible explanation for this complication may be the angiogenic properties of MSCs. There is experimental evidence that ADMSCs promote de novo formation of vascular structures and that these cells also secrete growth factors such as VEGF-A, VEGF-B, HIF-1α, and glial-derived neurotrophic factor (GDNF) under hypoxic conditions which could lead to endothelial cell network formation and trigger active neovascular growth [37–39].

Immunosuppression is still controversial and there is no established standard immunosuppressive therapy protocol after stem cell therapy. Among cell injection sites, the subretinal space is particularly advantageous as it is maintained as an immune privileged site by the connections between the RPE layer. Some authors believe that if the blood-retinal barrier is preserved during surgery, immunosuppressive drugs are not necessary. Thus, the success of subretinal transplantation depends on maintenance of RPE integrity. Moreover, both ESCs and MSCs have negligible immunogenicity, reducing the chance of rejection [40, 41]. On the other hand, some authors believe that there may be a disruption of the blood-retinal barrier by subretinal injection; the balance of the subretinal microenvironment may be broken and immunosuppression will be necessary until recovery of the barrier. There is also some evidence that suppression of the recipient’s immune response will increase the survival rate of transplanted cells [41]. Cyclosporin A is one of the most frequently used immunosuppressive drugs in ophthalmology. An animal study in retinal degenerative mice showed that cyclosporin A enhanced the survival of transplanted subretinal human retinal progenitor cells [42]. We preferred short-term cyclosporin A treatment for immunosuppression in our study. Further investigation is needed regarding immunosuppressive therapy in stem cell transplantation.

These are the limitations to our study. We know that the study population is small. Furthermore, the study included patients with poor visual function who are considered as legally blind which hindered our ability to perform reliable measurements of visual functions. Only one patient had an improvement in visual acuity (from 20/2000 to 20/400), perimetry, and ERG. Three patients mentioned that the light and some colors were brighter than before and there was a slight improvement in VA. The remaining seven patients had no VA improvement; six of them were only perception-positive before surgery. Visual field examinations were unreliable because of poor fixation and high false-negatives in nearly all of the participants. Patients with better VA are needed to determine the effect of this therapy on VA and the visual field. We observed small deposits on the inner aspects of Bruch’s membrane after surgery on OCT during the follow-up period. However, we do not know if these deposits are markers of donor cells, since ingestion of donor stem cells by host macrophages may produce a similar ophthalmoscopic appearance.

## Conclusion

Stem cell treatment with subretinal implantation of ADMSCs seems to have some ocular complications and should be applied with caution. The results of this study provide the first evidence of the short-term safety of subretinal implantation of ADMSCs in humans and clarifies the complications of the therapy which would be beneficial for future studies. To optimize the cell delivery technique and to evaluate the effects of this therapy on visual acuity and the quality of life of these patients, future studies including larger number of cases with better visual acuities will be necessary.

## Abbreviations

ADMSC: Adipose tissue-derived mesenchymal stem cell
AMD: Age-related macular degeneration
BCVA: Best corrected visual acuity
CNM: Choroidal neovascular membrane
DMEM: Dulbecco’s modified Eagle medium
ERG: Electroretinography
ERM: Epiretinal membrane
ESC: Embryonic stem cell
FFA: Fundus fluorescein angiography
GMP: Good manufacturing practice
IPSC: Induced pluripotent stem cell
ISCEV: International Society for Clinical Electrophysiology of Vision
MSC: Mesenchymal stem cell
NGF: Nerve growth factor
OCT: Optical coherence tomography
PBMC: Peripheral blood mononuclear cell
PBS: Phosphate-buffered saline
RP: Retinitis pigmentosa
RPE: Retina pigment epithelium
SMD: Stargardt macular dystrophy
VA: Visual acuity
VEGF: Vascular endothelial growth factor
